# Molecular Cloning and Functional Analysis of a Na^+^-Insensitive K^+^ Transporter of *Capsicum chinense* Jacq

**DOI:** 10.3389/fpls.2016.01980

**Published:** 2016-12-27

**Authors:** Nancy Ruiz-Lau, Emanuel Bojórquez-Quintal, Begoña Benito, Ileana Echevarría-Machado, Lucila A. Sánchez-Cach, María de Fátima Medina-Lara, Manuel Martínez-Estévez

**Affiliations:** ^1^Unidad de Bioquímica y Biología Molecular de Plantas, Centro de Investigación Científica de YucatánMérida, Mexico; ^2^CONACYT, Instituto Tecnológico Nacional de México, Instituto Tecnológico de Tuxtla GutiérrezTuxtla Gutiérrez, Mexico; ^3^CONACYT, Laboratorio de Análisis y Diagnóstico del Patrimonio, Colegio de MichoacánZamora, Mexico; ^4^Centro de Biotecnología y Genómica de Plantas, Universidad Politécnica de MadridMadrid, Spain

**Keywords:** *Capsicum chinense*, HAK-type transporter, K^+^-starvation, potassium, roots, sodium

## Abstract

High-affinity K^+^ (HAK) transporters are encoded by a large family of genes and are ubiquitous in the plant kingdom. These HAK-type transporters participate in low- and high-affinity potassium (K^+^) uptake and are crucial for the maintenance of K^+^ homeostasis under hostile conditions. In this study, the full-length cDNA of *CcHAK1* gene was isolated from roots of the habanero pepper (*Capsicum chinense*). *CcHAK1* expression was positively regulated by K^+^ starvation in roots and was not inhibited in the presence of NaCl. Phylogenetic analysis placed the CcHAK1 transporter in group I of the HAK K^+^ transporters, showing that it is closely related to *Capsicum annuum* CaHAK1 and *Solanum lycopersicum* LeHAK5. Characterization of the protein in a yeast mutant deficient in high-affinity K^+^ uptake (WΔ3) suggested that CcHAK1 function is associated with high-affinity K^+^ uptake, with K_m_ and V_max_ for Rb of 50 μM and 0.52 nmol mg^−1^ min^−1^, respectively. K^+^ uptake in yeast expressing the CcHAK1 transporter was inhibited by millimolar concentrations of the cations ammonium (${\text{NH}}_{4}^{+}$) and cesium (Cs^+^) but not by sodium (Na^+^). The results presented in this study suggest that the CcHAK1 transporter may contribute to the maintenance of K^+^ homeostasis in root cells in *C. chinense* plants undergoing K^+^-deficiency and salt stress.

## Introduction

Plants require a variety of mineral nutrients throughout their ontogeny to complete their growth and development (Marschner, 2012). Potassium (K^+^) is one of the most important essential macronutrients and the most abundant inorganic cation in plant cells. In plants, K^+^ represents 2–10% of the dry biomass and has a crucial role in many physiological and developmental processes, including adaptation to hostile conditions (Maathuis, 2009; Wang et al., 2013; Shabala and Pottosin, 2014). Despite the abundance of K^+^ in the earth's crust (2.1%), its low availability in the soil limits vegetal growth and reduces the productivity of large areas of arable land (Benito et al., 2014; Zörb et al., 2014). As a result, K^+^ fertilization has become a common and necessary practice in agriculture. However, such fertilization is very expensive, and a great proportion of the added K^+^ is lost by lixiviation (Römheld and Kirkby, 2010; Zörb et al., 2014).

The typical K^+^ concentration in the soil solution oscillates between 0.1 and 1 mM (Maathuis, 2009), but the concentration of available K^+^ is much lower. Consequently, plants have developed a variety of strategies, including symbiosis with microorganisms and specific K^+^ transport systems, to permit them to survive under conditions of low K^+^ availability (Garcia and Zimmermann, 2014; Nieves-Cordones et al., 2014). Potassium uptake in roots exhibits biphasic kinetics depending on the external concentration of K^+^, with components of low and high affinity (Epstein et al., 1963). High-affinity K^+^ uptake is an active process that is mediated primarily by transporters and passively by AKT1 (Arabidopsis K^+^ transporter 1) channel (Rubio et al., 2008; Pyo et al., 2010); whereas low-affinity K^+^ uptake is a passive process that occurs through membrane channels. The proteins that participate in the K^+^ transport and distribution processes in plants include AKT1 channels, high-affinity K^+^ transporters 2 (HKT2), cation/proton (H^+^) antiporters (CHX), and high-affinity K^+^ (HAK) symporters (Rodríguez-Navarro and Rubio, 2006; Rubio et al., 2010; Alemán et al., 2011; Ahmad and Maathuis, 2014; Chérel et al., 2014; Nieves-Cordones et al., 2014; Véry et al., 2014).

K^+^ uptake permeases/high-affinity K^+^ transporters/K^+^ transporters (KUP/HAK/KT) are represented by a large family of genes present in all biological kingdoms except *Animalia*; they are even found in viruses (Grabov, 2007; Greiner et al., 2011; Véry et al., 2014). HAK genes are present in all plant genomes studied to date, indicating the likely importance of these transporters in the sessile life style of plants (Rubio et al., 2000; Grabov, 2007; Véry et al., 2014). However, the number of HAK genes varies among species. At present, 13 such genes have been identified in *Arabidopsis thaliana* (Maser et al., 2001), 27 in *Oryza sativa* (Gupta et al., 2008; Yang et al., 2009), 27 in *Zea mays* (Zhang et al., 2012), 31 in *Populus trichocarpa* (He et al., 2012), 19 in *Solanum lycopersicum* (Nieves-Cordones et al., 2007; Hyun et al., 2014), and 16 in *Prunus persica* (Song Z. Z. et al., 2015). In addition, some members of the HAK gene family have been identified in *Capsicum annuum* (*CaHAK1*, Martínez-Cordero et al., 2004), *Hordeum vulgare* (HvHAK1-4, Santa-María et al., 1997; Vallejo et al., 2005; Boscari et al., 2009), *Lotus japonicus* (*LjKUP*, Desbrosses et al., 2004), *Vitis vinifera* (*VvKUP1-2*, Davies et al., 2006), *Gossypium hirsutum* (*GhKT1*, Ruan et al., 2001), in halophytes such as *Mesembryanthemum crystallinum* (*McHAK1-4*, Su et al., 2002), *Cymodocea nodosa* (*CnHAK1-2*, Garciadeblás et al., 2002), *Thellungiella halophila* (*ThHAK5*, Alemán et al., 2009), *Phragmites australis* (*PhaHAK1-2,5*, Takahashi et al., 2007a,b), *Aeluropus littoralis* (*AlHAK*, Su et al., 2007), and in the *Cryptomeria japonica* conifer (*CjKUP1*, Hosoo et al., 2014).

The transporters encoded by genes of the HAK family can be grouped into four (I–IV) phylogenetic groups (Rubio et al., 2000). At the transcriptional level, the members of this family are expressed in different tissues and organs (Su et al., 2002; Ahn et al., 2004; Yang et al., 2014; Song Z. et al., 2015) and are regulated by physiological conditions and environmental factors in a differential manner according to the group to which they belong (Véry et al., 2014; Song Z. et al., 2015). In particular, the genes that encode the HAK transporters of group I are positively regulated in roots under K^+^ starvation (Santa-María et al., 1997; Bañuelos et al., 2002; Ahn et al., 2004; Martínez-Cordero et al., 2004; Nieves-Cordones et al., 2007; Alemán et al., 2009; Horie et al., 2011; Shen et al., 2015), suggesting an adaptive role of group I transporters under conditions of low K^+^ availability. The positive regulation of the HAK genes by K^+^ deficiency can be inhibited by the presence of various ions. It has been reported that the cation sodium (Na^+^) prevents positive regulation by K^+^ starvation of *LeHAK5, AtHAK5*, and *ThHAK5* (Nieves-Cordones et al., 2007; Alemán et al., 2009), whereas the expression of *HvHAK1, OsHAK5, OsHAK21*, and some members of group II such as *McHAK1, McHAK3*, and *PhaHAK2* increases under these conditions (Su et al., 2002; Takahashi et al., 2007a; Fulgenzi et al., 2008; Yang et al., 2014; Shen et al., 2015). Ammonium (${\text{NH}}_{4}^{+}$) and cesium (Cs^+^) can induce the expression of HAK-type genes, suggesting the participation of HAK-type gene products in their transport and/or an effect of ${\text{NH}}_{4}^{+}$ and Cs^+^ on K^+^ levels through competitive inhibition (Nieves-Cordones et al., 2007; Qi et al., 2008; ten-Hoopen et al., 2010). Hormones such as abscisic acid (ABA) and ethylene, in addition to other factors such as membrane potential, osmotic stress, reactive oxygen species (ROS), and the processes of growth and stages of vegetal development of the plant, also regulate the expression of the HAK genes (see Véry et al., 2014).

The study of HAK transporters in heterologous systems such as bacteria, yeast and insect cells has provided crucial information on the function, selectivity, and affinity of the transport mediated by these proteins (Haro and Rodríguez-Navarro, 2003; Alemán et al., 2011). For example the expression in yeast mutants has shown that some members of HAK transporters that belong to group I display high affinity for K^+^ and poor discrimination between K^+^, Cs^+^, and rubidium (Rb^+^), and are inhibited by ${\text{NH}}_{4}^{+}$ and Na^+^ (Rubio et al., 2000; Martínez-Cordero et al., 2004; Alemán et al., 2009), whereas those of group II displayed high or low affinity for K^+^ and can even show dual affinities. The transporters of groups III and IV have been poorly studied, and their function as transporters is less well understood (Véry et al., 2014). Furthermore, HAK-type proteins mediates the transport of Cs^+^ under conditions of low K^+^ availability and to be inhibited by ${\text{NH}}_{4}^{+}$ (without transporting this cation), negatively correlating with the K^+^ uptake and content of cells (Santa-María et al., 2000; Nieves-Cordones et al., 2007; Qi et al., 2008; ten-Hoopen et al., 2010; Adams et al., 2013).

Recently, the participation of HAK transporters of group I in the maintenance of K^+^ homeostasis under hostile conditions has been reported (Nieves-Cordones et al., 2010, 2014; Alemán et al., 2014; Chérel et al., 2014; Yang et al., 2014; Shen et al., 2015). Salinity can induce K^+^ deficiency by inhibiting influx and increasing K^+^ efflux in roots, resulting in decreased K^+^ content of the plant (Bojórquez-Quintal et al., 2014; Demidchik, 2014; Shabala and Pottosin, 2014). In these adverse environmental conditions, the existence of a Na^+^-insensitive K^+^ uptake system in plant roots would undoubtedly be a useful strategy to maintain a high cytosolic K^+^/Na^+^ ratio crucial for salt tolerance (Shabala and Cuin, 2008). So far, only few candidates have been described like it is the rice OSHAK5 transporter. Its expression in the bright yellow 2 (BY2) tobacco cell line has demonstrated to increase the salinity tolerance of the cells (Horie et al., 2011), and overexpression of the same transporter in rice increased the K^+^/Na^+^ ratio and salt stress tolerance, suggesting the maintenance of high-affinity K^+^ uptake in the presence of Na^+^ (Yang et al., 2014). Also, AtHAK5 and OsHAK21 plays an important role in the absorption of K^+^ under conditions of K^+^ deficiency and high levels of Na^+^ (Nieves-Cordones et al., 2010; Shen et al., 2015).

*Capsicum chinense* (habanero pepper) is a species of pepper that is in great demand in Mexico and other countries due to its flavor, pungency, diversity in shape and fruit color (Bojórquez-Quintal et al., 2014). The production of habanero pepper fruits is directly related to K^+^ availability, and addition of this nutrient to the soil solution is necessary for their successful cultivation (Monforte-Gonzalez et al., 2010). However, the K^+^ fertilization seems not required for flowering, possibly due to the existence of efficient transport systems of K^+^ operating in this plant (Medina-Lara et al., 2008). In general, habanero pepper plants are cultivated in K^+^-rich soils, but a great proportion of this K^+^ is not available in the soil solution and cannot be absorbed by the plants (Borges-Gómez et al., 2005). Also, salinity problems could arise in these soils (Delgado et al., 2010) and induce K^+^ deficiency. Recent studies have suggested the presence of high- and low-affinity K^+^ transport mechanisms in the roots of the habanero pepper plants (Pacheco-Arjona et al., 2011). However, there is no information on the proteins that participate in K^+^ uptake in this species under conditions of K^+^ deficiency. In this work, we report the cDNA cloning of a HAK-type gene that is expressed in *C. chinense* roots. This gene, *CcHAK1*, is positively regulated by K^+^ deficiency and encodes a high-affinity K^+^ transporter (CcHAK1). Characterization of this transporter in *Saccharomyces cerevisiae* indicated that it mediates K^+^ uptake in the micromolar range and is insensitive to Na^+^.

## Materials and methods

### Plant material and growth conditions

Habanero pepper (*C. chinense* Jacq.) seeds (Chichen-Itza cultivar) (Seminis Vegetable Seeds, Inc., 2700 Camino del Sol, Oxnard, CA 93030, USA) were used in this study. The seeds were surface-sterilized with 80% ethanol (v/v) and sodium hypochlorite (30%, v/v, Cloralex™, North Alen, SA de CV) as described in Celis-Arámburo et al. (2011), washed with sterile water, pre-hydrated for 72 h in the dark at 4°C, and germinated by placing them in Petri dishes containing filter paper moistened with sterile grade milli-Q water. After germination, the seeds were transferred to containers with sterile vermiculite moistened with sterile milli-Q water, and the seedlings were grown for 45 days in a growth room at 25°C under a photoperiod cycle of 16/8 h light/dark and a light intensity of 123 μmol m^−2^ s^−1^. The seedlings were watered with sterile milli-Q water until the emergence of cotyledonal leaves (~15 days); then, a modified Hoagland nutrient solution (1/5 of the typical ionic strength) that contained the following micronutrients (μM) was applied: 50 CaCl_2_, 12.5 H_3_BO_3_, 1 MnSO_4_, 1 ZnSO_4_, 0.5 CuSO_4_, 0.1 (NH_4_)_6_Mo_7_O_24_, 0.1 NiCl, 10 Fe-EDTA, and macronutrients (mM): 1.2 KNO_3_, 0.8 Ca(NO_3_)_2_, 0.2 KH_2_PO_4_, 0.2 MgSO_4_. The nutrient solution was replaced with fresh solution each week until 45 days of plants growth.

Potassium starvation experiments were conducted under hydroponic conditions; 45-day-old seedlings, at which the vermiculite was removed, were transferred to a nutrient solution lacking K^+^ but otherwise containing the micronutrients described above. The macronutrient concentrations were modified to the following concentrations (in mM): 0.1 Ca(H_2_PO_4_)_2_, 1.4 Ca(NO_3_)_2_, 0.2 MgSO_4_, without K^+^. The nutrient solution was replaced twice during the experiment. Roots from seedlings growing in the absence of K^+^ were harvested at 15-days, quickly frozen in liquid nitrogen and stored at −80°C until total RNA extraction was performed.

### Isolation, sequence analysis of *CcHAK1* cDNA, and phylogenetic tree

Total RNA was isolated from root tissue using a NucleoSpin RNA Plant Kit (Macherey-Nagel, Neumann-Neander Str. 6–8, Düren, Germany). cDNA was synthesized from 1 μg of total RNA using commercial avian myeloblastosis virus reverse transcriptase (First-strand cDNA Synthesis Kit, Amersahm Biosciences, Freiburg Germany) with an anchored oligo-dT primer according to the manufacturer's instructions. To obtain an initial fragment containing the habanero pepper *HAK1* gene, the reverse transcription products were amplified by the polymerase chain reaction (PCR) using Taq high-fidelity polymerase (Roche) and degenerate primers previously described by Martínez-Cordero et al. (2004) to isolate a *HAK1* gene from *C. annuum*. The PCR product was cloned into the pCR2.1-TOPO vector using a TA Cloning Kit (Invitrogen, Carlsbad, CA, USA) and sequenced. The missing portions of the cDNA at the 5′ and 3′ ends were rescued by rapid amplification of 5′ and 3′ cDNA ends (5′/3′ RACE kit 2nd generation, Roche, Mannheim, Germany) following the manufacturer's instructions. The full-length cDNA, which was designated *CcHAK1*, was obtained after PCR using the sense primer 5′-GTCTAGAAAACAATGGCTAGCTCAGATAGTGAT-3′ and the antisense primer 5′-CGAATTCGTTATACCTCATAAGTCATGCCAACC-3′ that included the initiation codon ATG and a stop codon, respectively. These primers included Xba I and EcoR I restriction sites appropriate for insertion of *CcHAK1* in the sense orientation into a yeast expression vector. The sequence of the primer containing the initiation codon ATG was modified according to Hamilton et al. (1987) and Haro et al. (2013) to enhance protein expression in yeast. PCR products were cloned into the pCR2.1-TOPO vector using a TA Cloning Kit (Invitrogen) and verified by sequencing.

Protein and nucleotide alignments were obtained using the Clustal W2 program (http://www.ebi.ac.uk) and BLAST (http://www.ncbi.nlm.nih.gov/BLAST/) in *tblastp* form. The phylogenetic tree was generated using the neighbor-joining method with MEGA software. The molecular weight was determined by the program Compute Pi/Mw tool (http://expasy.org/). Hydrophobicity parameters were calculated by TopPred-Topology prediction of membrane protein (http://mobyle.pasteur.fr/cgi-bin/portal.py?=toppred), TMpred (http://www.ch.embnet.org/software/TMPRED_form.html), TMHMM (http://www.cbs.dtu.dk/services/TMHMM/). A hypothetical model for the membrane topology of the protein encoded by the amplified cDNA (CcHAK1) was built using the TMHMM server (http://www.cbs.dtu.dk/services/TMHMM/).

### Functional complementation of CcHAK1 in the yeast *saccharomyces cerevisiae*

The pCR2.1-TOPO + *CcHAK1* cDNA construct was digested with *Xba I* and *EcoR I* restriction enzymes, and a 2421-bp fragment containing the *CcHAK1* open reading frame (ORF, 2415 bp in length) plus 5 bp at the 5′-proximal ATG codon and 1 bp at the 3′-terminal STOP codon (AAACA and C, respectively, to enhance protein expression in yeast) was ligated into the yeast expression vector pYPGE15 (Brunelli and Pall, 1993) to generate the construct pYPGE15+CcHAK1. For functional complementation experiments, pYPGE15 (the parent plasmid) and pYPGE15+CcHAK1 were transformed into K^+^ uptake- deficient yeast (*S. cerevisiae*) strain WΔ3 (*MATa, ade2, ura3, trp1, trk1*Δ*::*LEU2 *trk2*Δ*::*HIS3; Haro et al., 1999; Haro and Rodríguez-Navarro, 2003). The yeast strains transformed with the plasmid pYPGE15 containing the *CcHAK1* cDNA and with the empty plasmid were designated as WΔ3-CcHAK1 and WΔ3-p, respectively. The WΔ3 strains were maintained in yeast extract-peptone-dextrose (YPD) medium and minimal medium SD (Sherman, 1991) supplemented with uracil (appropriate nutritional requirements according to the auxotrophic markers) and 50 mM K^+^.

Complementation assays growth of yeast at low K^+^ were performed in Petri dishes containing solid arginine phosphate medium lacking uracil (AP-U) (Rodríguez-Navarro and Ramos, 1984) supplemented with concentrations of K^+^ ranging from 0.05 to 5 mM, in the absence or presence of 20 mM NH_4_Cl, 5 mM CsCl or various concentrations of NaCl (50 and 100 mM), for inhibition studies. For growth, the yeast strains were incubated at 28°C. In some cases, WΔ3-CaHAK1 (WΔ3 strain transformed with *CaHAK1*, the *HAK1* gene of *C. annuum* (Martínez-Cordero et al., 2004) was used as a positive control for the yeast growth in the micromolar concentrations of K^+^.

### Growth curves of yeast transformants

The WΔ3-CcHAK1strain was grown in liquid AP-U medium at 28°C under continuous agitation and supplemented with various concentrations of K^+^ (0.05, 0.1, and 5 mM K^+^) or with 0.1 mM K^+^ plus 10 mM NaCl. A Bioscreen C Microbiology Reader (OY Growth Curve AB Ltd., Helsinki, Finland) was used to measure the optical densities of the cultures every 3 h for 3 consecutive days of growth at 28°C in continuous agitation. In this assay, the WΔ3-CaHAK1 strain was used as a positive control. The experiment was repeated thrice.

### Cation uptake experiments in yeast

To evaluate the K^+^ transport capacity of CcHAK1 and to determine the values of K_m_ and V_max_, two tests were performed: measurement of K^+^ depletion from the culture medium and measurement of cation accumulation in the cells. For both assays, yeast cells were grown overnight at 28°C in AP-U medium (supplemented with 50 mM K^+^ for WΔ3-pYPGE15 and WΔ3-p) and then starved of K^+^ for 4 h in K^+^-free AP-U medium. The cells were then suspended in 10 mM MES supplemented with 2% glucose and adjusted to pH 6 with Ca(OH)_2_. At time zero, the indicated concentrations of cations (KCl or RbCl) were added to the medium and the samples were collected at intervals over a 2-h period. The experiment was repeated thrice.

For K^+^ depletion experiments, the cells were suspended in AP-U medium containing various micromolar concentrations of K^+^ plus 1 mM CsCl, 1 mM NH_4_Cl, or 5 mM NaCl for inhibition studies. Samples of the medium (1 ml) were removed at various time intervals and centrifuged at 5000 rpm for 1 min, and the K^+^ concentration in the supernatant was measured. For K^+^ uptake assays, 5- or 10-ml samples were taken at intervals, filtered through an AAWP nitrocellulose membrane filter (0.8-μm pore, Millipore, Molsheim, France) and washed with 20 mM MgCl_2_. The filters were incubated overnight in 0.1 M HCl and 10 mM MgCl_2_. The results are expressed on a cell dry weight basis. Data from three independent experiments for each condition were fitted to the Michaelis-Menten equation. Cations were identified and quantified by atomic emission spectrophotometry using a Perkin-Elmer Model 2380 spectrophotometer (Norwalk, CT, USA; Fraile-Escanciano et al., 2010). Control experiments were performed with the WΔ3 strain transformed with plasmid without an insert (WΔ3-p).

### Analysis of transcript levels of *CcHAK1* by RT-PCR

To evaluate CcHAK1 expression, 45-day-old habanero pepper seedlings were transferred to a modified Hoagland nutrient solution containing 1.4 mM K^+^ (KCl, control) or 50 μM K^+^ (deficit) with 0, 10, 30, or 50 mM NaCl for 10 days. Semi-quantitative PCR was performed using cDNA synthesized from 1 μg of total RNA isolated as described above. For PCR, we used sense (5′-TACAACAACAAGTGGATTCAAG-3′) and antisense (5′-CGAATTCGTTATACCTCATAAGTCATGCCAACC-3′) primers designed based on the total cDNA sequence from *CcHAK1*. PCR was conducted after a 5 min denaturation step at 95°C followed by 25, 28, 30, or 35 cycles of 30 s at 95°C, 30 s at 55°C, and 40 s at 72°C. Tubulin served as a positive control in the reaction with primers forward (5′-GACCTTGAATCGGCTTATGG-3′) and reverse (5′ TATCCTGGGTGAACGCTTTG 3′). RT-PCR was performed with two different RNA extracts from leaves and roots tissues of each treatment. PCR was repeated three times using *Taq* polymerase (Sigma).

### Statistical analysis

To yeast growth curves and cation uptake experiments the results are representative of three independent experiments. Date are subjected to analysis of variance (ANOVA) and mean comparisons were made using Tukey's multiple range test (*P* ≤ 0.05), using SIGMA STAT v.12.

## Results

### Isolation and sequence analysis of *CcHAK1* cDNA

Total RNA was isolated from the roots of *C. chinense* seedlings exposed to K^+^ starvation for 15 days to identify putative K^+^ transporters that can be expressed in this growth conditions. Using degenerated primers designed from HAK transporters by Martínez-Cordero et al. (2004), a fragment an 843-bp region was initially amplified. The full-length cDNA was obtained by extension of the 5′ and 3′ ends using the 5′/3′ RACE Kit. The final clone obtained had an insert of 2415 bp that contained the *CcHAK1*.

**Figure 1:**
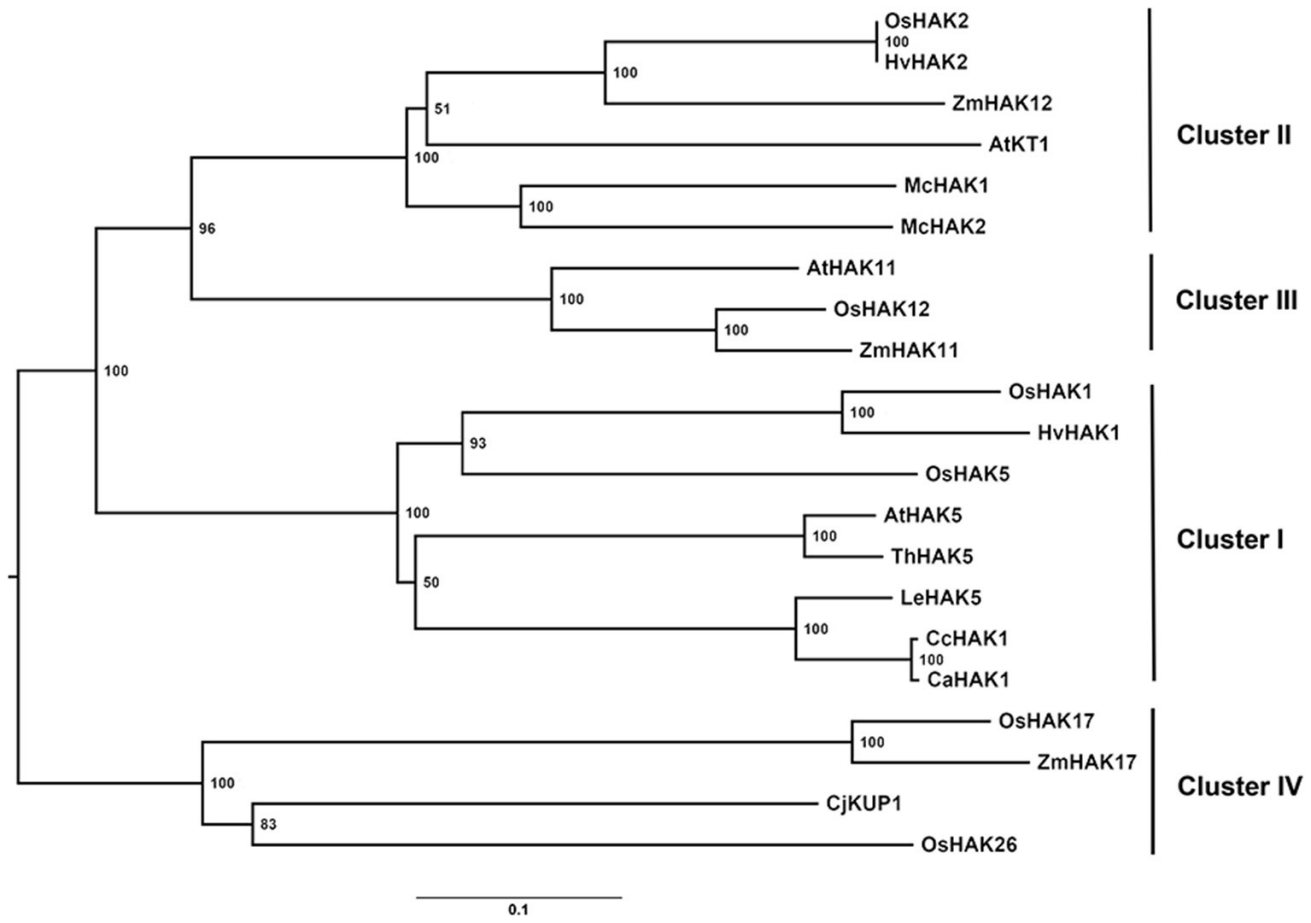
**Phylogenetic tree of the proteins of the KUP/HAK/KT transporter family**. The most representative members of each group of HAK transporters and the CcHAK1 transporter were used in the phylogenetic study. The tree was constructed using the nearest-neighbor algorithm with MEGA software, and the bookstrap values from 1000 replicates are shown at each node. The accession numbers are as follows: AtHAK5 (AF129478), AtHAK11 (BT002147.1), AtKT1 (AF012656.1), CaHAK1 (AY560009), CcHAK1 (KT202302), CjKUP1 (AB915694), HvHAK1 (AF025292), HvHAK2 (AF129479.1), LeHAK5 (DQ489721), McHAK1 (AF367864.1), McHAK2 (AF367865.1), OsHAK1 (AJ427970), OsHAK2 (AK070575), OsHAK5 (AK241580), OsHAK12 (AJ427981.1), OsHAK17 (AJ427975.1), OsHAK26 (AK072472), ThHAK5 (EF177193), ZmHAK11 (DAA36040.1), ZmHAK12 (AFW56980.1), ZmHAK17 (DAA61709.1).

*In silico* analysis of the *CcHAK1* sequence revealed that it encodes a polypeptide of 804 amino acids with a predictive molecular mass of 89.86 kDa (Figures S1A, S2). The CcHAK1 putative protein showed characteristic conserved regions, such as the **G**VIY**GD**IGT**SPLY** sequence (the conserved amino acids are shown in bold), common to transporters of the KUP/HAK/KT family (Figures S1A, S2; Rubio et al., 2000). Hydrophobicity analysis of the CcHAK1 sequence predicted the presence of 12 transmembrane regions and a long carboxyl-terminal tail (Figure S1). Alignment of the CcHAK1 sequence with the sequence of other transporters (ClustalW2) gave 99% similarity with CaHAK1 and 85% with SlHAK5. Furthermore, a difference of only eight amino acids between the CcHAK1 and CaHAK1 sequences was observed (Figure S2). Phylogenetic analysis placed CcHAK1 protein in group I of the K^+^ transporters, close to CaHAK1 and SlHAK5 (Figure 1). Interestingly, unlike most HAK transporters of group I (Figure 1), the CcHAK1 sequence shows a change in only one amino acid at the N356T position (Figure S2), where there is a threonine (T) instead of an asparagine (N).

### *CcHAK1* encodes a high-affinity K^+^ transporter

**Figure 2:**
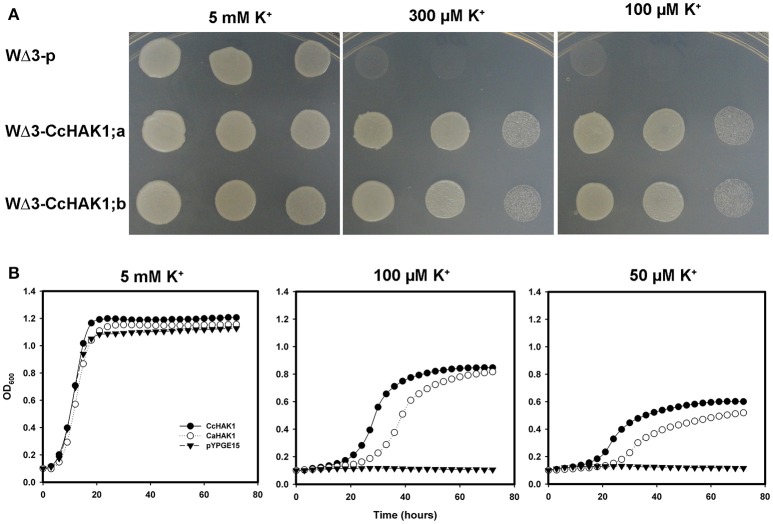
**CcHAK1 complementation assay in yeast cells deficient in high-affinity K^+^ uptake. (A)** Growth of the mutant strain of WΔ3 yeast in solid arginine phosphate medium lacking uracil (AP-U) supplemented with various concentrations of K^+^. The mutant yeast strain was transformed with the empty plasmid pYPGE15 (WΔ3-p) or with the plasmid containing the *CcHAK1* cDNA (WΔ3-CcHAK1;a and WΔ3-CcHAK1;b, independent clones). Drop serial dilutions of the cell culture were inoculated on agar plates containing AP-U medium. **(B)** Growth curves of the WΔ3 strain transformed with the empty plasmid (closed triangles), *CcHAK1* (closed circles), or *CaHAK1* (open circles) in liquid medium (AP-U) supplemented with 5 mM, 100 μM, or 50 μM K^+^.

All HAK-type transporters of group I that have been characterized to date are regulated by the absence of K^+^ in the medium and show a high affinity for the cation, especially when the availability of external K^+^ is low, with K_m_ values (Rb^+^) in the micromolar range (Rodríguez-Navarro and Rubio, 2006; Grabov, 2007; Véry et al., 2014). To determine whether CcHAK1 functions as a K^+^ transporter, the *CcHAK1* cDNA was cloned in the yeast expression vector pYPGE15, and the construction was transformed into the WΔ3 yeast strain, which is deficient in high-affinity K^+^ uptake systems (Haro and Rodríguez-Navarro, 2003). In the presence of 5 mM K^+^, the growth of the WΔ3-CcHAK1 transformants were similar to that of the yeast strain transformed with the empty vector (WΔ3-p; Figure 2A). However, at 300 and 100 μM, only the expression of CcHAK1 could restored the growth of the WΔ3 mutant. In contrast, growth of the mutant transformed with the empty plasmid (WΔ3-p) under the same conditions was not observed (Figure 2A). These results suggest that CcHAK1 is involved in high-affinity K^+^ transport.

For a more precise study of the growth recovery capacity of the WΔ3 mutants, growth curves of the cells in liquid arginine phosphate lacking uracil (AP-U) medium at various concentrations of K^+^ were performed (Figure 2B). For this study, the growth of transformants expressing CcHAK1 and its *C. annuum* CaHAK1 counterpart were compared (Martínez-Cordero et al., 2004). At 5 mM K^+^, the three transformed strains showed similar growth. However, WΔ3-p failed to grow at micromolar concentrations of K^+^. On the other hand, the transformants expressing the HAK1 transporter grew even in the presence of 50 μM K^+^. These results indicate that CcHAK1 expression enables growth of the WΔ3 mutant at micromolar concentrations of K^+^, suggesting that it confers high-affinity transport of K^+^ like its previously characterized CaHAK1 counterpart (Martínez-Cordero et al., 2004).

**Figure 3:**
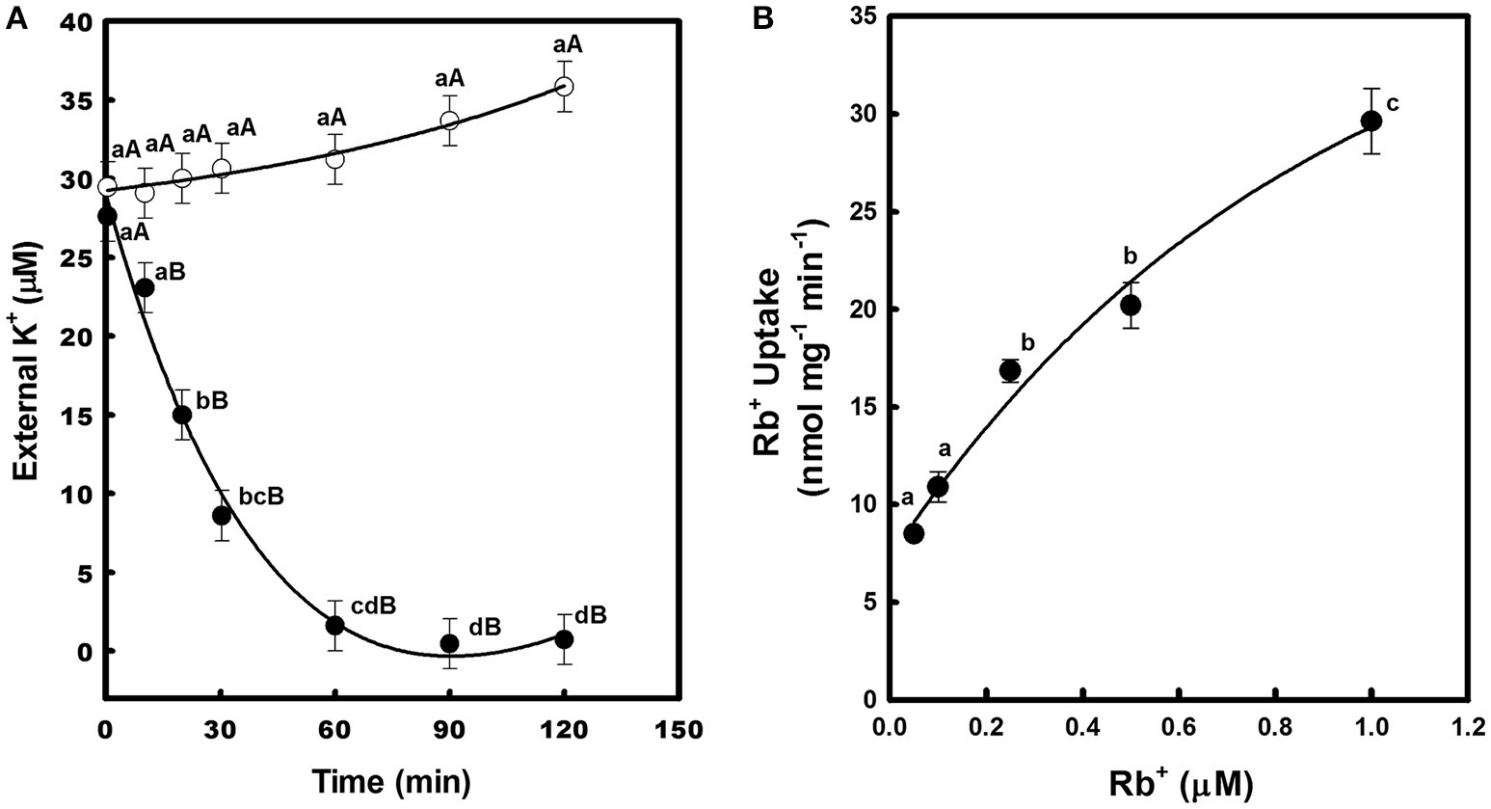
**Depletion of external K^+^ and kinetics of Rb^+^ uptake in cell suspensions of yeast expressing the CcHAK1 transporter. (A)** K^+^ uptake in yeast. WΔ3 strains transformed with *CcHAK1* cDNA (WΔ3-CcHAK1, closed circles) or with the empty plasmid (WΔ3-p, open circles) were subjected to K^+^ starvation for 4 h. At time zero, 25 μM K^+^ (KCl) was added to the suspension solution, which consisted of 10 mM MES pH 6, supplemented with glucose at 2%. The concentration of K^+^ was measured in the medium at intervals over a 2-h period. **(B)** Rb^+^ uptake in yeast. Rb^+^ adsorption values at various external concentrations of Rb^+^ are shown. The data were fitted to the Michaelis–Menten equation; a K_m_ of 50 μM and a V_max_ of 0.52 nmol mg^−1^ min^−1^ for Rb^+^ were calculated. The WΔ3 strain transformed with the *CcHAK1* cDNA was subjected to K^+^ starvation for 4 h and suspended in 10 mM MES at pH 6 for Rb^+^ uptake experiments. Figures show the data of a representative experiment of three repetitions. Data are mean ± SE of three replicates. Different lowercase letters represent significant differences (*p* ≤ 0.05; Tukey's test) within a strain between time points **(A)** or Rb^+^ concentrations **(B)**, while different capital letters **(A)** represent significant differences (*p* ≤ 0.05; Tukey's test) between strains within the same day.

To confirm that CcHAK1 is a K^+^ transporter, a kinetic study of the depletion of K^+^ from the medium was performed (Figure 3A). The WΔ3-CcHAK1 yeasts depleted the K^+^ present in the external medium (25 μM K^+^) after 60 min. No depletion of K^+^ was observed in medium containing the WΔ3-p strain, indicating that the observed K^+^ uptake from the WΔ3-CcHAK1 medium was due to the expression of CcHAK1 (Figure 3A). Kinetic characterization of the transport mediated by CcHAK1 was carried out using Rb^+^ in the absorption experiments. The Rb^+^ is a K^+^ analogue that is commonly used in kinetic analyses; CcHAK1 does not discriminate between these two cations (data not shown). CcHAK1 mediates high-affinity Rb^+^ uptake with an apparent K_m_ of 50 μM and a V_max_ of 0.52 nmol mg^−1^ min^−1^ (Figure 3B).

### Effects of ${\text{NH}}_{4}^{+}$, Cs^+^, and Na^+^ on the transport of K^+^

**Figure 4:**
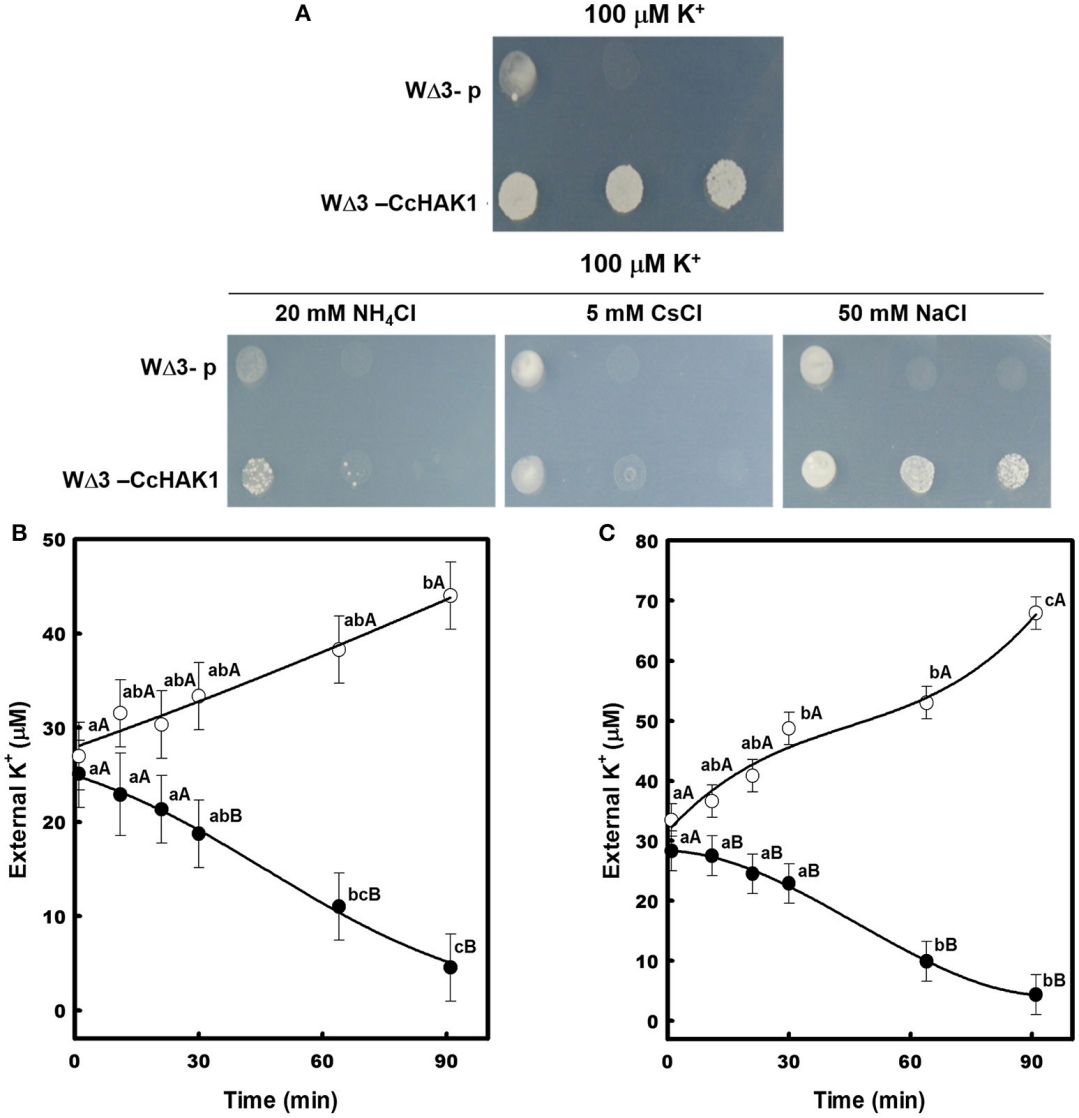
**Effects of NH_4_Cl, CsCl, and NaCl on the high-affinity K^+^ uptake mediated by CcHAK1 in the yeast mutant. (A)** Growth of yeast transformed with *CcHAK1* cDNA (WΔ3-CcHAK1) in the presence of NH_4_Cl, CsCl, and NaCl. WΔ3-p: empty plasmid. **(B)** K^+^ uptake in the presence (open circles) and absence (closed circles) of 1 mM NH_4_Cl. **(C)** High-affinity K^+^ uptake in the presence (open circles) and absence (closed circles) of 1 mM CsCl. The WΔ3 strain transformed with *CcHAK1* cDNA was subjected to K^+^ starvation for 4 h prior to the beginning of the experiment. The depletion of K^+^ was measured at intervals over a 90-min period. One representative experiment (of three) is shown. Figures show the data of a representative experiment of three repetitions. Data are mean ± SE of three replicates. Different lowercase letters represent significant differences (*p* ≤ 0.05; Tukey's test) within a treatment between times, while different capital letters represent significant differences (*p* ≤ 0.05; Tukey's test) between treatments within the same day.

To determine the effect of ${\text{NH}}_{4}^{+}$, Cs^+^, and Na^+^ on the transport of high-affinity K^+^, drop complementation assays and K^+^ depletion studies were performed. Growth of the WΔ3-CcHAK1 strain was inhibited when ${\text{NH}}_{4}^{+}$ and Cs^+^ were added to the culture medium (AP-U). In contrast, the growth of CcHAK1 transformants was insensitive to Na^+^ (Figure 4A). WΔ3 yeast cells expressing *CcHAK1* cDNA were capable of depleting the external K^+^ in the culture medium after 90 min under conditions of ${\text{NH}}_{4}^{+}$ absence. The presence of 1 mM NH_4_Cl inhibited the transport of K^+^ (Figure 4B). Similar results were observed using 1 mM CsCl (Figure 4C). An increase in external K^+^ concentration was observed for WΔ3-CcHAK1 after 60 min of treatment with ${\text{NH}}_{4}^{+}$ (Figure 4A) and Cs^+^ (Figure 4C).

**Figure 5:**
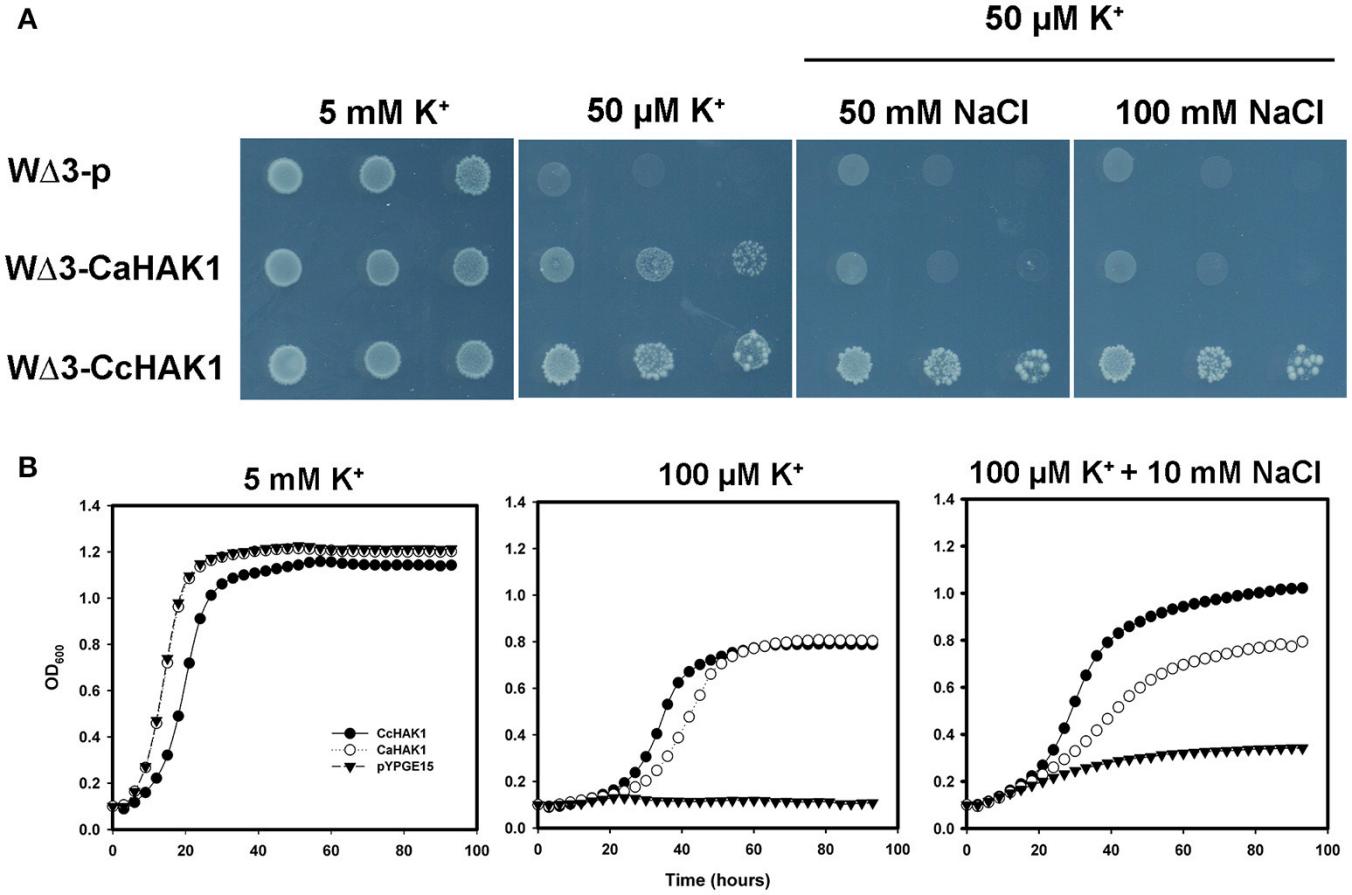
**Growth of yeast transformed with *CcHAK1* (WΔ3-CcHAK1) or *CaHAK1* (WΔ3-CaHAK1) cDNA in the presence of NaCl. (A)** Drop complementation assay in solid arginine phosphate medium lacking uracil (AP-U) supplemented with 50 μM K^+^ and various concentrations of NaCl. WΔ3-p: transformant harboring the empty plasmid. **(B)** Growth curves of the WΔ3 strain transformed with the empty pYPGE15 plasmid (closed triangles), *CcHAK1* (closed circles), or *CaHAK1* (open circles) in liquid medium (AP-U) supplemented with 100 μM K^+^ in the presence or absence of 10 mM NaCl. As a control for optical density, WΔ3 cells were inoculated with 5 mM K^+^.

To corroborate the insensitivity to Na^+^ of the K^+^ transport observed in the WΔ3-CcHAK1 transformants, a drop complementation assay was performed in which the growth of the CaHAK1 transformant sensitive to Na^+^ (Martínez-Cordero et al., 2004) was compared with that of the CcHAK1 transformant (Figure 5). AP-U medium containing 50 μM KCl was supplemented with various concentrations of NaCl. The WΔ3-CcHAK1 yeast strain grew in the presence of 50 and 100 mM NaCl, whereas growth of the CaHAK1-transformed strains and the empty plasmid were inhibited in the presence of NaCl. The latter strain was incapable of growing at 50 μM K^+^. All transformants showed similar growth at 5 mM K^+^ (Figure 5A).

**Figure 6:**
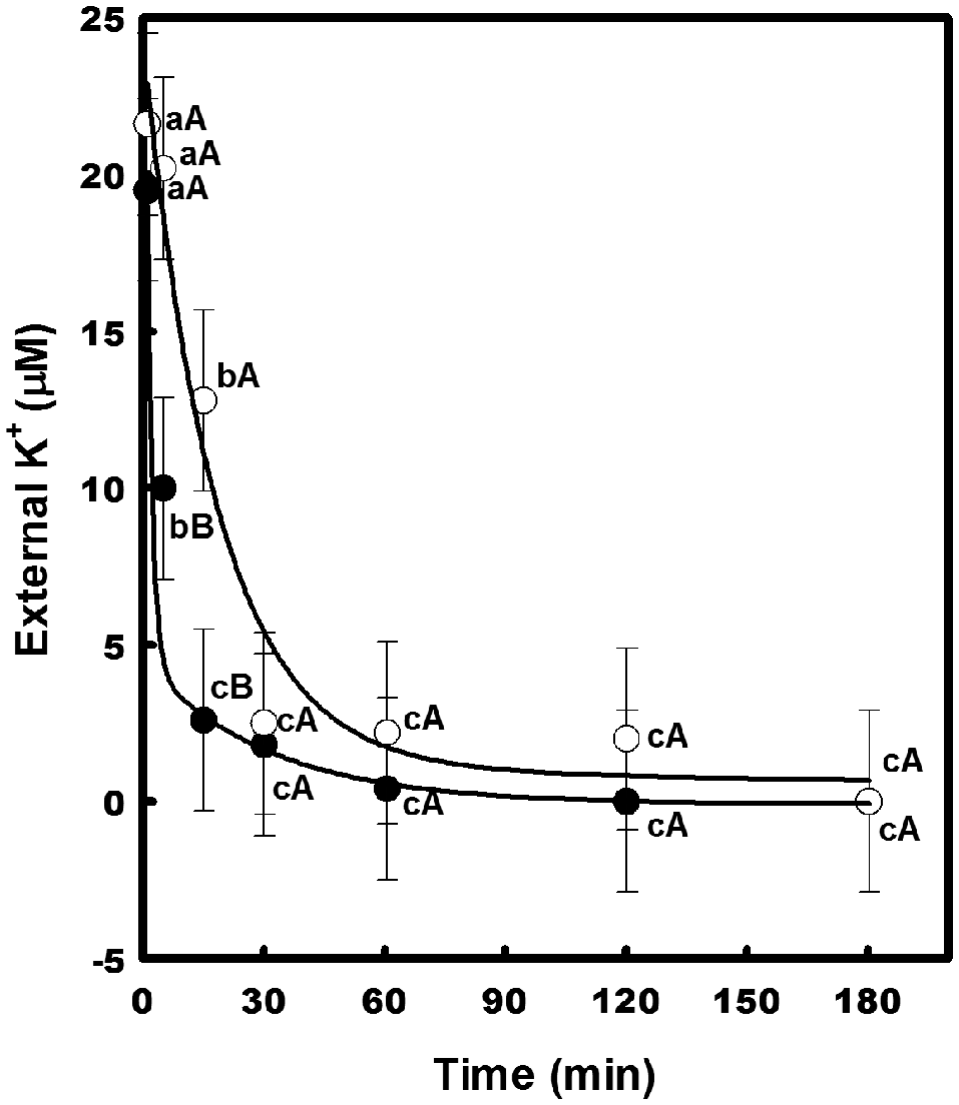
**Effect of NaCl on the high-affinity K^+^ uptake mediated by CcHAK1**. High-affinity K^+^ uptake in the presence (open circles) and absence (closed circles) of 5 mM NaCl is shown. The WΔ3 strain transformed with *CcHAK1* cDNA was subjected to K^+^ starvation for 4 h prior to the beginning of the experiment. The depletion of K^+^ was measured at intervals over a 2–3 h period. Figure show the data of a representative experiment of three repetitions. Data are mean ± SE of three replicates. Different lowercase letters significant differences (*p* ≤ 0.05; Tukey's test) within a treatment between time points, while different capital letters represent significant differences (*p* ≤ 0.05; Tukey's test) between treatments within the same time.

The effect of Na^+^ on the growth of the transformants was evaluated in liquid medium (AP-U) supplemented with 100 μM K^+^ in the presence or absence of 10 mM NaCl (Figure 5). Unlike the WΔ3-p strain, the growth of the WΔ3-CaHAK1, and WΔ3-CcHAK1 transformants showed no inhibition by Na^+^ at low concentrations of K^+^ until 96 h. However, the growth of the yeast mutants expressing CaHAK1 was much slower than that of the WΔ3-CcHAK1 mutant (Figure 5B). The depletion of external K^+^ by the WΔ3-CcHAK1 strain was not inhibited by the presence of NaCl, but it displayed slower kinetics than were observed in the absence of NaCl (Figure 6).

### Semi-quantitative expression of CcHAK1 in pepper roots

**Figure 7:**
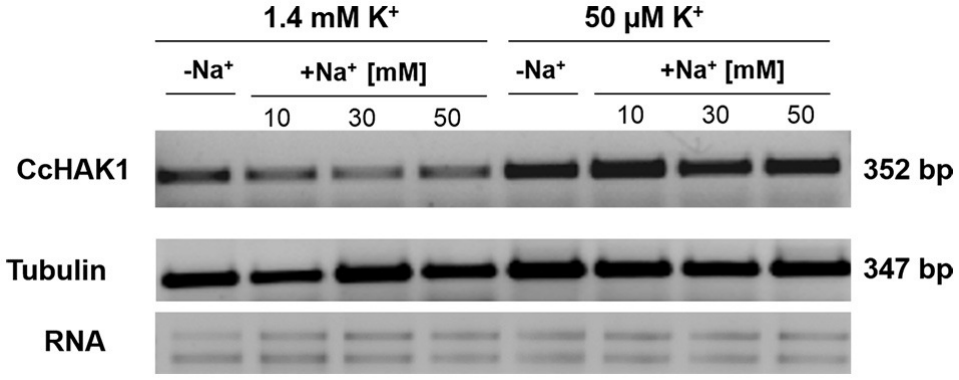
**Effects of K^+^ and NaCl on CcHAK1 transcript levels**. Habanero pepper seedlings 45 days old were transferred for 10 days to Hoagland solution [1/5] containing 1.4 mM or 0.05 mM K^+^ in the absence or presence of 10, 30, or 50 mM NaCl. The transcript levels were evaluated by RT-PCR using the tubulin gene as a loading control.

The expression of the HAK genes is regulated by environmental conditions, stage of vegetal development and factors such as ions, hormones, and ROS (see Véry et al., 2014). To address this question, an analysis of *CcHAK1* expression in habanero pepper roots under various growth conditions was performed using semi-quantitative PCR (Figure 7). *CcHAK1* transcripts were detected in normal plant growth conditions (1.4 mM K^+^ in the growth medium) in leaves and roots, its expression was induced mainly in the roots of seedlings exposed to K^+^ deprivation, and its expression was maintained even in the presence of 50 mM NaCl (Figure 7 and Figure S3).

## Discussion

In this study, the cDNA corresponding to the *CcHAK1* gene (KT202302) was isolated from the RNA of habanero pepper roots (*C. chinense*) grown in the absence of K^+^. The *CcHAK1* cDNA was 2415 bp in length. This gene encodes a HAK-type high-affinity K^+^ transporter of 804 amino acids (CcHAK1) with a predicted molecular mass of 89.86 KDa. CcHAK1 presents all of the structural characteristics that have previously been reported for HAK-type transporters (Figure S1), including 12 transmembrane domains, a long loop between the second and third transmembrane segments and a long carboxyl end (Rodriguez-Navarro, 2000; Gómez-Porras et al., 2012). These transporters belong to a large family of K^+^ transporter genes known as KUP/HAK/KT transporters that are present in non-animal cells and are ubiquitous in plants (Grabov, 2007; Greiner et al., 2011; Véry et al., 2014). Genome sequencing and molecular cloning projects have resulted in the identification of genes of the HAK family in various plant species (Santa-María et al., 1997; Maser et al., 2001; Garciadeblás et al., 2002; Su et al., 2002; Desbrosses et al., 2004; Davies et al., 2006; Takahashi et al., 2007a,b; Alemán et al., 2009; Yang et al., 2009; He et al., 2012; Zhang et al., 2012; Hosoo et al., 2014; Hyun et al., 2014; Song Z. Z. et al., 2015). In Solanaceae, 19 genes have been identified in tomato, including *LeHAK5* (Nieves-Cordones et al., 2007; Hyun et al., 2014) and a member in pepper, *CaHAK1* (Martínez-Cordero et al., 2004). The *CcHAK1* cDNA is the second HAK-type gene discovered in peppers and the first in the *C. chinense* species; it shows 99% identity with *CaHAK1* and 84% with *LeHAK5*.

The phylogenetic analysis showed that CcHAK1 belonged to group I of the HAK transporters (Rubio et al., 2000) and that it is closely related to the *C. annuum* CaHAK1 and the *S. lycopersicum* LeHAK5 (Figure 1). In addition to the high degree of homology between CcHAK1 and CaHAK1, both proteins consist of 804 amino acids, and they differ in only eight amino acid residues (Figure S2). One of these changes in amino acid residues is of particular interest because is one of the most conserved in group I of HAKs (Figure 1). CcHAK1 has a threonine (T) instead of an asparagine (N) at the 356 position (T356N) (Figure S2). Both T and N are polar, hydrophilic amino acids, but they differ in the lengths of their lateral chains. OsHAK5, which is a group I K^+^ transporter, has a histidine (H) at the H362N position (Figure S2). It has been reported that this HAK-type transporter shows regulatory characteristics that differ from those of other members of the group (Horie et al., 2011; Yang et al., 2014). Point mutations in the HAK transporters can modify its affinity for K^+^ and its sensitivity to Na^+^, as has been reported for HvHAK1 and AtHAK5 (Mangano et al., 2008; Alemán et al., 2014). Recent studies have identified the amino acid residues in transmembrane regions and loops of *E. coli* HAK transporters that are critical for K^+^ uptake by these proteins (Sato et al., 2014). These findings suggest that CcHAK1 could possess characteristics that differ from those of other members of group I of the HAK family. In future studies, a directed mutagenesis approach on specific amino acids in CcHAK1 should offer interesting results.

In this study, CcHAK1 function was characterized in the WΔ3 yeast strain, which is defective in high-affinity K^+^ uptake (Haro and Rodríguez-Navarro, 2003). CcHAK1 expression complemented the growth of the WΔ3 yeast strain and depleted the external K^+^ (μM) present in the medium (Figures 2, 3A). This result demonstrates that CcHAK1, mediates high-affinity K^+^ uptake. Expression of CcHAK1 in yeast showed that it has an apparent K_m_ for Rb^+^ of 50 μM and a V_max_ of 0.52 nmol mg^−1^ min^−1^ (Figure 3B). It is worth noting that the CcHAK1 K_m_ (Rb^+^) is ~26-fold higher than that of its CaHAK1 homologous (1.9 μM) (Martínez-Cordero et al., 2004). The difference in the K_m_ values suggests that *C. chinense* possesses low K^+^ uptake capacity under low K^+^ availability conditions. However, cultures of the WΔ3-CcHAK1 strain achieved higher optical density values than the transformants that expresses CaHAK1 at low concentrations of K^+^ (Figure 2B). In general, CcHAK1 has one of the highest K_m_ (Rb^+^) values of the HAK-type transporters of group I that have been studied to date. Also, according to the expression analysis carried out in this study, *CcHAK1* transcripts were detected in roots of plants grown in K^+^ normal conditions although its expression was enhanced during K^+^ deprivation (Figure 7). This expression pattern has been described previously in HvHAK1 (Santa-María et al., 1997) and in AtHAK5 (Rubio et al., 2000).

In roots and heterologous systems that express HAK-type proteins, high-affinity K^+^ uptake is inhibited by ${\text{NH}}_{4}^{+}$, Cs^+^, and Na^+^ (Véry et al., 2014). As has been reported for CaHAK1 (Martínez-Cordero et al., 2004), the growth of the WΔ3-CcHAK1 strain and its high-affinity K^+^ uptake were inhibited at millimolar concentrations of ${\text{NH}}_{4}^{+}$ and Cs^+^ (Figure 4). These data agree with the results of previous studies of pepper roots in which ${\text{NH}}_{4}^{+}$ and Cs^+^ were found to competitively inhibit K^+^ uptake at low concentrations (Martínez-Cordero et al., 2005; Pacheco-Arjona et al., 2011). In different plant species, two components of high-affinity K^+^ uptake have been identified: a component that is sensitive to ${\text{NH}}_{4}^{+}$ and is mediated by HAK-type transporters and a component that is insensitive to ${\text{NH}}_{4}^{+}$ and is mediated by AKT1-type K^+^ channels (Spalding et al., 1999; Santa-María et al., 2000; Martínez-Cordero et al., 2005; Nieves-Cordones et al., 2007; Pacheco-Arjona et al., 2011). Cs^+^ induces K^+^ deficiency in cells by inhibiting K^+^ uptake through AKT1 channels and HAK-type transporters under conditions of both high and low K^+^ availability (Hampton et al., 2004; Qi et al., 2008; Adams et al., 2013). In the current study, the K^+^ transport mediated by CcHAK1 is inhibited by both ${\text{NH}}_{4}^{+}$ and Cs^+^.

The increase of K in the medium of growing for W3-CcHAK1 strain in the presence of ${\text{NH}}_{4}^{+}$ and Cs^+^ can indicate K leakage from the cells. We suggested that in yeast, the uptake of these cations can causes a membrane depolarization which drives to an activation of outwardly rectifying plasma membrane potassium channel Tork1. Tork1 is the only potassium-specific efflux system described in yeast and its activity is regulated by membrane potential (Yenush, 2016).

Different responses to the Na^+^ effect have been reported for HAK-type transporters (Véry et al., 2014) but remarkably only a few examples, like OsHAK5, have demonstrated to be Na^+^-insensitive K^+^ uptake systems. In our work, CcHAK1 expression complemented the growth of the strain at low concentrations of K^+^ and in the presence of NaCl (Figure 4A) and in spite of the high sequence homology this is a functional difference between CaHAK1 and CcHAK1 (Figure 5). CaHAK1 like AtHAK5, OsHAK1, and HvHAK1 are group I transporters whose high-affinity K^+^ uptake is sensitive to Na^+^, probably due to a competitive inhibition mechanism (Rubio et al., 2000; Bañuelos et al., 2002; Martínez-Cordero et al., 2004; Fulgenzi et al., 2008). In fact, HvHAK1 and other transporters such as PhaHAK5, PhaHAK2, and OsHAK2 can mediate uptake of Na^+^ present at the millimolar level and inhibit K^+^ uptake (Santa-María et al., 1997; Takahashi et al., 2007a,b). In this study, the Na^+^ uptake capacity of the strain that expresses CcHAK1 was not determined, but Na^+^ did not interfere with K^+^ uptake (Figure 6). OsHAK5 is an atypical transporter that mediates K^+^ uptake insensitive to Na^+^. In *E. coli* and BY2 cells, the OsHAK5 transporter preferentially accumulates K^+^ rather than Na^+^ under NaCl stress (Horie et al., 2011). Similarly, CcHAK1 was shown to be Na^+^-insensitive; and the presence of Na^+^ merely made the high-affinity K^+^ uptake lightly slow (Figure 6). Surprisingly, in both CcHAK1 and OsHAK5, the N residue corresponding to positions 356 and 362 of the respective protein sequences is substituted with another amino acid, suggesting that this amino acid residue may participate in the regulation of the Na^+^ effect (Figure S2). Also, the HAK transporters of some halophytic species are insensitive to Na^+^, and this has been related to their tolerance of salinity (Garciadeblás et al., 2002; Su et al., 2007; Takahashi et al., 2007b). There are few studies about the effect of salinity on habanero pepper, which has been classified as a glycophyte (Niu and Rodríguez, 2010; Niu et al., 2010). Nevertheless, this specie is widely cultivated in the Yucatan coast, México, where the saline intrusion has increased, due to an intensification of the agricultural land use during recent years that has caused that soil's electrical conductivity to reach maximum values of 3.21 dS m^−1^ (Delgado et al., 2010).

Similar to all HAK-type genes of group I and some members of group II (Véry et al., 2014), the expression of *CcHAK1* mainly in roots is regulated by K^+^ deficiency but it was also expressed under control conditions (Figure 7 and Figure S3). This finding suggests that the transporter that encodes *CcHAK1* has an adaptive role under conditions of low K^+^ availability. On the other hand, the presence of Na^+^ in the medium can affect the expression of the genes that encode HAK-type transporters, especially under K^+^ deficiency conditions (Véry et al., 2014). Unlike the case with *AtHAK5, LeHAK5*, and *ThHAK5* (Nieves-Cordones et al., 2007; Alemán et al., 2009), Na^+^ did not decrease *CcHAK1* expression under K^+^ deficiency or control conditions (Figure 7). Considering the insensitivity of CcHAK1 expression to Na^+^, the results suggest a possible role for this protein in maintaining K^+^ homeostasis in root cells under saline stress. Other genes that encode HAK transporters of group II show various levels of expression in the presence of high concentrations of Na^+^. *PhaHAK2, McHAK1*, and *McHAK3*, which are genes of halophyte species, are positively regulated by Na^+^ (Su et al., 2002; Takahashi et al., 2007a). In barley, the accumulation of *HvHAK1* transcripts temporarily increases in the presence of Na^+^ (Fulgenzi et al., 2008). However, K^+^ uptake through the PhaHAK2 and HvHAK1 transporters is sensitive to high concentrations of Na^+^ (Takahashi et al., 2007a; Fulgenzi et al., 2008; Véry et al., 2014). In rice roots, hulls and vascular tissues, the levels of *OsHAK5* and *OsHAK21* transcripts are positively regulated by high concentrations of NaCl under conditions of low and high K^+^ availability (Yang et al., 2014; Shen et al., 2015).

Leakage of K^+^ from root cells is a common response that occurs in the presence of NaCl due to depolarization of the plasma membrane (Demidchik et al., 2014). Also, other ROS-activated mechanisms may contribute to this K^+^-efflux in some species (Bose et al., 2014). This depolarization makes K^+^ uptake through AKT1 channels thermodynamically impossible and conditions the plants to take up K^+^ through HAK-type transporters (Anschütz et al., 2014; Demidchik et al., 2014). It has recently been reported that the AtHAK5, OsHAK5, and OsHAK21 transporters are required for growth and K^+^ uptake under NaCl stress (Nieves-Cordones et al., 2010; Yang et al., 2014; Shen et al., 2015). Overexpression of *OsHAK5* in rice and BY2 cells improves the salt tolerance of the cells by increasing the K^+^/Na^+^ ratio (Horie et al., 2011; Yang et al., 2014). *Capsicum* is a genus that is moderately sensitive to saline stress throughout its ontogeny with a significant reduction in fruit production (Bojórquez-Quintal et al., 2012). In habanero pepper, NaCl induces K^+^ efflux and reduces the K^+^ content of roots at high salt stress (Bojórquez-Quintal et al., 2014). The results regarding *CcHAK1* expression and high-affinity K^+^ uptake in the presence of Na^+^ presented in this work suggest that, in habanero peppers, continuous high-affinity K^+^ uptake may occur to maintain the K^+^/Na^+^ ratio under saline stress. In fact, the content of K^+^ in habanero pepper roots is maintained at low and moderate concentrations of NaCl (Bojórquez-Quintal et al., 2014). The successful cultivation and production of habanero pepper fruits is directly related to K^+^ availability (Monforte-Gonzalez et al., 2010) and salt stress induces the leakage of K^+^. Taking into account that maintenance of K^+^ absorption and decrease in Na^+^ accumulation represents an important strategy in developing tolerance to saline stress (Shabala and Cuin, 2008) and that the habanero pepper (*C. chinense*) actually is grown intensively in the southern region of Mexico where salinity problems could arise in the future (Delgado et al., 2010), the overexpression of Na^+^-insensitive K^+^ transporters such as CcHAK1 provides an attractive alternative for the improvement of glycophyte species production such as the peppers and to enhance salt tolerance of plants.

## Accession number

The nucleotide sequence reported in this paper has been submitted to GenBank with accession number KT202302.

## Author contributions

MM: Group leader and head of the research project; IE proposed experiments and writing manuscript; EB, NR, LS, BB, MFM, Isolation, sequence analysis of CcHAK1 cDNA, and phylogenetic tree. Functional complementation of CcHAK1 in the yeast *Saccharomyces cerevisiae*. Analysis of transcript levels of CcHAK1 by RT-PCR. Cation uptake experiments in yeast.

## Funding

This work was supported by Consejo Nacional de Ciencia y Tecnología (CONACYT) project # 166621-Z.

### Conflict of interest statement

The authors declare that the research was conducted in the absence of any commercial or financial relationships that could be construed as a potential conflict of interest.

## Abbreviations

AKT1: Arabidopsis K^+^ transporter 1
AP-U medium: arginine phosphate lacking uracil medium
BY2 cells: bright yellow 2 cells
CHX: cation/proton (H^+^) antiporters
HAK: high-affinity K^+^ transporter
HKT2: high-affinity K^+^ transporters 2
KUP/HAK/KT: K^+^ uptake permeases/high-affinity K^+^ transporters/K^+^ transporter
ORF: open reading frame
YPD medium: yeast extract-peptone-dextrose.
