# Supplementary material for: Molecular Cloning and Functional Analysis of a Na+-Insensitive K+ Transporter of Capsicum chinense Jacq
Source: Front Plant Sci. 2016 Dec 27;7:1980. doi: 10.3389/fpls.2016.01980 (PMC5186809; doi:10.3389/fpls.2016.01980)
Supplement: Supplementary file 1 [file DataSheet1.DOC]

**(A)**

**MASSDSDHHTDQEIVNGGQLKDRKVSWAKLARVDSLNLEAGKVSSTPENHNSTADWKTVLSLAFQSVGVIYGDIGTSPLYVFASTFTDKIGHKDDILGVLSLIIYTIILVPMTKYVFIVLWANNNGDGGAFALYSLLCRYAKVSLIPNQEPEDRELSHYSLDIPSNHIRRAQRIRHSLEKSKFAKIFLVFLAILGTSMVIGDGVLTPCISVLSAVSGIKPLGQEAVVGISVAILIALFCAQRFGTDKVGYTFAPAICIWFMFISGIGLYNLFKYDVSVLRAFNPKYLINYFQRNGRKGWISLGGVFLCITGSEAMFADLGHFSVRSIQISFSCLVFPALLSAYSGQAAYLSKFPETVSNTFYDSVPDPLYWPTFVVAVAAAIIASQAMISGTFSIVAQAQSIGCFPRVKVVHTSPKHGGQVYIPELNYFLMIACVIVILSFKTTEKLGHAYGIAVVSAEIITTHMVTLVMLVIWKTRIWWITLFYGTYLFIESTYFSAQLTKFTQGGYLPIAFSVVLVIIMGTWHYVQKLRYQFELSNKVSSEYIRDLANNPDIKRVRGIGLLYSELVQGIPPIFHHFVSNIPSVHSVIVLVSIKSIPISKVALQERFLFRHVEPREYKVFRCVVRLGYKDQLGDTANFENQLVEQLNKFIRHEHYILAAQEQVLADRETEPASGQLVPGRSSKVHIEEDLQQQVDSRISTSTRSIHSVHTPTAQSNRSSSRTQMVPPNASGQEEMQFVEKAKEQGVFYLLAEAEVVAKKDSSFVKKAFVNYGYNFLRKNFRQGEKVMAIPQTRLLRVGMTYEV**

| **PROGRAMA** | **I** | **II** | **III** | **IV** | **V** | **VI** | **VII** | **VIII** | **IX** | **X** | **XI** | **XII** |
| --- | --- | --- | --- | --- | --- | --- | --- | --- | --- | --- | --- | --- |
| Toppred | 67-87 | 95-115 | 183-203 | 221-241 | 248-268 | 297-317 | 326-346 | 369-389 | 424-444 | 456-476 | 506-526 | 574-594 |
| TMpred | 56-83 | 95-120 | 183-201 | 220-240 | 252-272 | 298-317 | 327-347 | 369-389 | 426-444 | 447-474 | 508-524 | 578-594 |
| TMHMM | 62-84 | 97-119 | 186-205 | 226-245 | 250-272 | 298-317 | 327-349 | 369-391 | 421-440 | 452-474 | 478-500 | 507-526 |

**(B)**


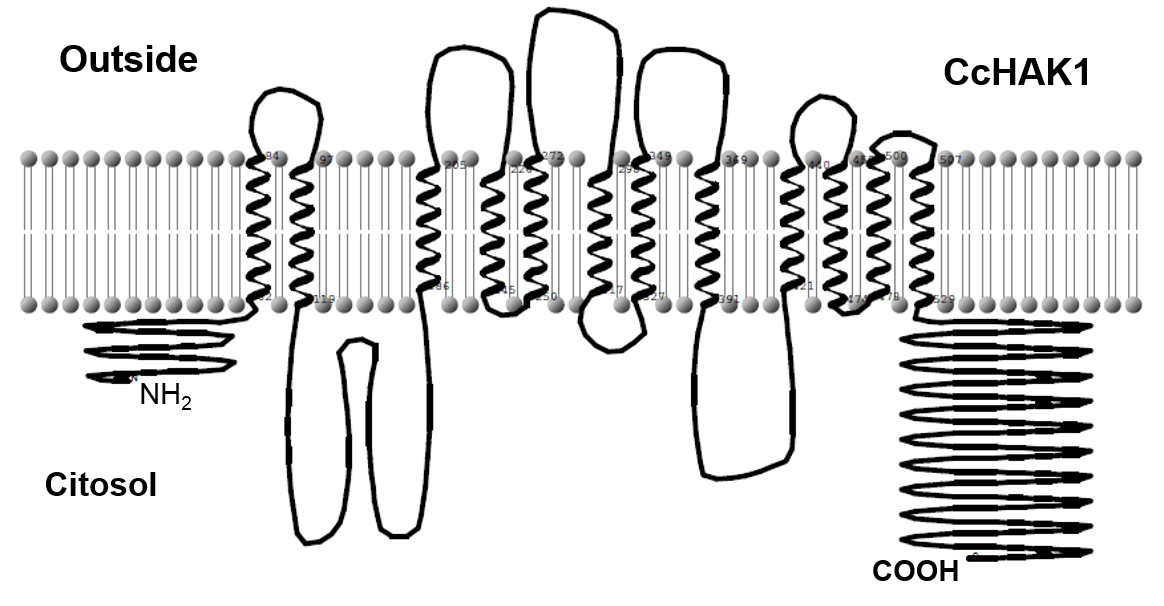


**Fig. S1** Hydrophobicity profile of the CcHAK1 protein. (A) Predicting the limits of the transmembrane segments of CcHAK1. The limits of the segments are shaded gray (TMHMM). The conserved sequence in the first transmembrane segment was outlined and the conserved amino acids in all HAK-type transportes are shown in bold. The amino acid residue in the 356 position for CcHAK1 is in red. Programs: TopPred (<http://mobyle.pasteur.fr/cgi-bin/potal.py?Form=toppred>), TMpred (<http://www.ch.embnet.org/software/TMPRED_form.html>), TMHMM (<http://www.cbs.dtu.dk/services/TMHMMM/>). (B) Proposed topology for the CcHAK1 transporter developed using the TMHMM server.

**(A)**

CcHAK1 MASSDSDHHTDQE----**I**VNG--------GQLKDRKVSWAKLARVDSLNLEAGKVSSTPE 48

CaHAK1 MASSDSDHHTDQE----**V**VNG--------GQLKDRKVSWAKLARVDSLNLEAGKVSSTPE 48

LeHAK5 MESTKSEEEVN-------VGQ--------QQLKDRKVSWAKLGRVDSLNMEAGKVSSTQA 45

AtHAK5 --MDGEEHQIDGD----EVNN------HENKLNEKKKSWGKLYRPDSFIIEAGQTPTNTG 48

ThHAK5 --MDGEEHQIDG-----EVNNQEHNHDHEHKLKEKKKSWGKLFRPDSFSIEAGKTPKNTG 53

OsHAK5 --MTEPLHTSSNG----GAER---GPNAAFESEKTLQTTTRLQRFDSLHMEAGKIPGGQS 51

OsHAK1 --MSSALEVEGSGSPGV--------------EPAATATASRLKRHDSLFGDAEKVSGGKH 44

HvHAK1 --MS--LQVE---------------------DPRSAETPAPLKRHDSLFGDAEKVSDSKH 35

. : * * **: :* : .

CcHAK1 NHNSTADWKTVLSLAFQSV**G**VIY**GD**IGT**SPLY**VFASTFTDKIGHKDDILGVLSLIIYTII 108

CaHAK1 NHNSTADWKTVLSLAFQSV**G**VIY**GD**IGT**SPLY**VFASTFTDKIGHKDDILGVLSLIIYTII 108

LeHAK5 RHGSKGDWKTILSLAFQSV**G**VIY**GD**IGT**SPLY**VFASTFTDEIKHKDDILGVLSLIIYTIM 105

AtHAK5 -RRSLMSWRTTMSLAFQSL**G**VVY**GD**IGT**SPLY**VYASTFTDGINDKDDVVGVLSLIIYTIT 107

ThHAK5 -HSSLLSWRTTMSLAFQSL**G**VVY**GD**IGT**SPLY**VYASTFTEGINDKDDVIGVLSLIIYTLT 112

OsHAK5 -HTAKVGWATTLHLAFQSI**G**VVY**GD**MGT**SPLY**VFSSTFTNGIKDTNDILGVMSLIIYTVV 110

OsHAK1 HGGSAVSWAVTLHLAFQSV**G**IIY**GD**IGT**SPLY**VYSSTFPDGIGHRDDLVGVLSLILYTLI 104

HvHAK1 HG-SQVSWMRTLSLAFQSV**G**IIY**GD**IGT**SPLY**VYSSTFPDGIKNRDDLLGVLSLILYTLI 94

: .* : *****:*::***:*******::***.: * . :*::**:***:**:

CcHAK1 LVPMTKYVFIVLWANNNGDGGAFALYSLLCRYAKVSLIPNQEPEDRELSHYSLDIPSNHI 168

CaHAK1 LVPMTKYVFIVLWANNNGDGGAFALYSLLCRYAKVSLIPNQEPEDRELSHYSLDIPSNHI 168

LeHAK5 LVPMTKYVFIVLWANDNGDGGAFALYSLLCRYAKVSLIPNQQPEDRELSHYSLDLPSNHI 165

AtHAK5 LVALLKYVFIVLQANDNGEGGTFALYSLICRYAKMGLIPNQEPEDVELSNYTLELPTTQL 167

ThHAK5 LVALLKYVFIVLQANDNGEGGTFALYSLICRYAKTGLIPNQEPEDSELSNYTLELPNTKI 172

OsHAK5 LLPLIKYCFIVLRANDNGDGGTFALYSLISRYARISLIPNQQAEDAMVSHYKLESPSNRV 170

OsHAK1 IIPMLKYVFIVLYANDNGDGGTFALYSLISRYAKIRMIPNQQAEDAMVSNYSIEAPSSQL 164

HvHAK1 IIPMLKYVFIVLYANDNGDGGTFALYSLISRYAKIRLIPDQQAEDAAVSNYHIEAPNSQL 154

::.: ** **** **:**:**:******:.***: :**:*:.** :*:* :: *..::

CcHAK1 RRAQRIRHSLEKSKFAK**I**FLVFLAILGTSMVIGDGVLTPCISVLSAVSGIKP----LGQE 224

CaHAK1 RRAQRIRHSLEKSKFAK**F**FLVFLAILGTSMVIGDGVLTPCISVLSAVSGIKP----LGQE 224

LeHAK5 KRAQRIRQGLEKSKFAKIFLVFLAILGTSMVIGDGVLTPCISVLSAVSGIKP----LGQD 221

AtHAK5 RRAHMIKEKLENSKFAKIILFLVTIMGTSMVIGDGILTPSISVLSAVSGIKS----LGQN 223

ThHAK5 RRSHKIKEKLENSKFAKIILFLVTIMGTSMVIGDGILTPSISVLSAVSGIKS----LGQN 228

OsHAK5 KRAHWIKEKMENSPNFKIILFLVTILATSMVIGDGVLTPCISVLSAVGGIKESAKSLTQG 230

OsHAK1 RRAQWVKHKLESSRAAKMALFFLTILGTSMVMGDGTLTPAISVLSAVSGIREKAPNLTQT 224

HvHAK1 KRAQWLKQKLESSKAAKIVLFTLTILGTSMVIGDGTLTPAISVLSAVSGIREKAPSLTQT 214

:*:: ::. :*.* *: *. ::*:.****:*** ***.*******.**: * *

CcHAK1 AVVGISVAIL**I**ALFCAQRFGTDKVGYTFAPAICIWFMFISGIGLYNLFKYDVSVLRAFNP 284

CaHAK1 AVVGISVAIL**V**ALFCAQRFGTDKVGYTFAPAICIWFMFISGIGLYNLFKYDVSVLRAFNP 284

LeHAK5 AIMGISIAILVILFSLQRMGTDKVGYTFAPAICVWFLFISGIGLYNLFKYDVTVLRAFNP 281

AtHAK5 TVVGVSVAILIVLFAFQRFGTDKVGFSFAPIILVWFTFLIGIGLFNLFKHDITVLKALNP 283

ThHAK5 TVVGVSVAILILLFAFQRFGTDKVGFSFAPIIFVWFMFLTGIGLVNLFKHDITVLKALNP 288

OsHAK5 QIAGIAIAILIVLFLVQRFGTDKVGYSFGPIILTWFIFIAGTGVYNLFKHDTGVLKAFNP 290

OsHAK1 QVVLISVAILFMLFSVQRFGTDKVGYTFAPIISVWFLLIAGIGLYNLVVHEITILKAFNP 284

HvHAK1 QVVLISVAILFMLFSVQRFGTDKVGYTFAPVISVWFLLIAGIGMYNLVVHDIGVLRAFNP 274

: :::***. ** **:******::*.* * ** :: * *: **. :: :*:*:**

CcHAK1 KYLINYFQRNG**R**KGWISLGGVFLCITGSEAMFADLGHFSVRSIQISFSCLVFPALLSAYS 344

CaHAK1 KYLINYFQRNG**K**KGWISLGGVFLCITGSEAMFADLGHFSVRSIQISFSCLVFPALLSAYS 344

LeHAK5 MYIIHYFKRNGKKGWISLGGVFLCITGSEAMFADLGHFSVRSIQISFSCLVFPALLSAYS 341

AtHAK5 LYIIYYFRRTGRQGWISLGGVFLCITGTEAMFADLGHFSVRAVQISFSCVAYPALVTIYC 343

ThHAK5 LYIIHYFRRNGKKGWISLGGVFLCITGTEAMFADLGHFSVRAVQISFSCIAYPALVTIYC 348

OsHAK5 KYIVDYFERNGKQGWISLGGVILCITGTEAMFADLGHFNVRAIQIGFSVVLLPSVLLAYI 350

OsHAK1 WYIVQYFRRNGKKGWVSLGGVVLCVTGTEGMFADLGHFNIRAVQISFNCILFPSVALCYI 344

HvHAK1 MYIVQYFIRNGKSGWVSLGGIILCVTGTEGMFADLGHFNIRAVQLSFNGILFPSVALCYI 334

*:: ** *.*:.**:****:.**:**:*.********.:*::*:.*. : *:: *

CcHAK1 GQAAYLSKFPE**T**VSNTFYDS**V**PD---------PLYWPTFVVAVAAAIIASQAMISGTFSI 395

CaHAK1 GQAAYLSKFPE**N**VSNTFYDS**L**PD---------PLYWPTFVVAVAAAIIASQAMISGTFSI 395

LeHAK5 GQAAYLTKFPE**N**VANTFYDCIPG---------PLYWPTFVVAVAAAIIASQAMISGTFSI 392

AtHAK5 GQAAYLTKHTY**N**VSNTFYDSIPD---------PLYWPTFVVAVAASIIASQAMISGAFSV 394

ThHAK5 GQAAYLTKHTS**N**VSNTFYDSIPD---------PFYWPTFVVAVAASIIASQAMISGAFSV 399

OsHAK5 GQAAYLRIYPE**H**VADTFYKSIP--------------------VAAAIIASQAMISGAFAI 390

OsHAK1 GQAAYLRKFPE**N**VSDTFYKSIPGKYRDRLNFGPLFWPTFIVAILAAIIASQAMLSGAFAI 404

HvHAK1 GQAAYLRKFPD**N**VANTFYRSIP---------APMFWPTFIVAILAAIIASQAMLSGAFAI 385

****** .. *::*** .:* : *:*******:**:*::

CcHAK1 VAQAQSIGCFPRVKVVHTSPKHGGQVYIPELNYFLMIACVIVILSFKTTEKLGHAYGIAV 455

CaHAK1 VAQAQSIGCFPRVKVVHTSPKHGGQVYIPELNYFLMIACVIVILSFKTTEKLGHAYGIAV 455

LeHAK5 VAQAQNVGCFPRVKVIHTSTKHDGQVYIPELNYFLMIACVLVTLSFKTTEKLGHAYGIAV 452

AtHAK5 ISQSLRMGCFPRVKVVHTSAKYEGQVYIPEINYLLMLACIAVTLAFRTTEKIGHAYGIAV 454

ThHAK5 ISQSLRMGCFPRVKVVHTSAKYEGQVYIPEINYFLMLACVAVTLTFRTTEKIGHAYGIAV 459

OsHAK5 IAQSQILGCFPRVRVIHTSTKFHGQVYIPEINYVLMVLCVAVTAIFQTTDKIGNAYGIAV 450

OsHAK1 LSKALSLGCLPRVRVIHTSKKYEGQVYIPEVNFMMGLASIIVTIAFRTTTSIGNAYGICV 464

HvHAK1 LSKALSLGCMPRVRVIHTSHKYEGQVYIPEVNFLMGLASIVVTVAFRTTTSIGHAYGICV 445

:::: :**:***:*:*** *. *******:*:.: : .: * *:** .:*:****.*

CcHAK1 VSAEIITTHMVTLVMLVIWKTRIWWITLFYGTYLFIESTYFSAQLTKFTQGGYLPIAFSV 515

CaHAK1 VSAEIITTHMVTLVMLVIWKTRIWWITLFYGTYLFIESTYFSAQLTKFTQGGYLPIAFSV 515

LeHAK5 VSAEIITTHMVTLVMLVIWKTKIWWITLFYAVYLSIESTYFSAQLTKFTQGGYLPMAFSV 512

AtHAK5 VTVMVITTLMVTLIMLVIWKTNIVWIAIFLVVFGSIEMLYLSSVMYKFTSGGYLPLTITV 514

ThHAK5 VTVMVITTFMVTLIMLVIWKTNIVWIAMFLIGFGSIEMLYLSSVMYKFTSGGYLPLAITL 519

OsHAK5 VFVMFITTLLVTLVMVMIWKTSLLWIALFPVIFGGAELIYLSSAFYKFTQGGYLPLVFSA 510

OsHAK1 VTTFMVTTHLMTVVMLLIWKKHLVFILLFYCVFGFTEVVYLSSILSKFVDGGYLPFCFAM 524

HvHAK1 VTTFAITTHLMTVVMLLIWKKHVMFIMLFYVVFGSIELIYLSSIMSKFIEGGYLPICFAL 505

* . :** ::*::*::***. : :* :* : * *:*: : ** .*****: ::

CcHAK1 VLVIIMGTWHYVQKLRYQFELSNKVSSEYIRDLANNPD-IKRVRGIGLLYSELVQGIPPI 574

CaHAK1 VLVIIMGTWHYVQKLRYQFELSNKVSSEYIRDLANNPD-IKRVRGIGLLYSELVQGIPPI 574

LeHAK5 VLVIIMGTWYYVQKLRYEFELNNKVSTEYISDLANNPD-IKRVPGIGLLYSELVQGIPPI 571

AtHAK5 VLMAMMAIWQYVHVLKYRYELREKISRENAIQMATSPD-VNRVPGIGLFYTELVNGITPL 573

ThHAK5 VLMAMMAIWQYVHVLKYRYELREKISGENAIQMATSPN-VNRVPGIALFYTELVHGITPL 578

OsHAK5 ILMFIMATWHYVHVHRYKYELRNKVSNNYVAELAVKQN-LARLPGIGFLYSELVQGIPPI 569

OsHAK1 VLMTMMATWHYVHVRRYWYELDHIVPTAELASLLEENGGVRRVPGVGLLYTELVQGIPPL 584

HvHAK1 VVMSLMAAWHYVQVRRYWYELDHIVPISEMTMLLEKNE-VRRIPGVGLLYTELVQGIPPV 564

::: :*. * **: :* :** . :. : . : *: *:.::*:***:**.*:

CcHAK1 FHHFVSNIPSVHSVIVLVSIKSIPISKVALQERFLFRHVEPREYKVFRCVVRLGYKDQLG 634

CaHAK1 FHHFVSNIPSVHSVIVLVSIKSIPISKVALQERFLFRHVEPREYKVFRCVVRLGYKDQLG 634

LeHAK5 FPHFVSNIPSVHSVIVLVSIKSIPISKVALQERFLFRHVEPREYKVFRCVVRLGYKDQLG 631

AtHAK5 FSHYISNLSSVHSVFVLISIKTLPVNRVTSSERFFFRYVGPKDSGMFRCVVRYGYKEDIE 633

ThHAK5 FSHYISNLSSVHSVFVLISIKSLPVSRVTPSERFFFRYMEPKDCGMFRCVVRYGYKEDIE 638

OsHAK5 LPHLVEKVPSIHSVLVIISIKYLPISKIETKERFLFRYVEPKEYRVFRCVVRYGYNDKVE 629

OsHAK1 FPRLVRKIPSVHAVFVFISIKHLPIPHVAAAERFLFRQVGPRARRVFRCVARYGYTDALE 644

HvHAK1 FPRLIQKIPSVHSIFIFMSIKHLPISRVVPTERFIFRQVGPREHRMFRCVARYGYSDTLE 624

: : : ::.*:*::::::*** :*: :: ***:** : *: :****.* **.: :

CcHAK1 DTANFENQLVEQLNKFIRHEHYILAAQEQVLA**D**RETEPASGQLVPGRSSKVHIEEDLQQQ 694

CaHAK1 DTANFENQLVEQLNKFIRHEHYILAAQEQVLA**E**RETEPASGQLVPGRSSKVHIEEDLQQQ 694

LeHAK5 DTMDFENQLVEQLNKFIRHEHYILEAHEQVVNR------------EKTSRVHIEEEMEQP 679

AtHAK5 EPDEFERHFVYYLKEFIHHEHFMSGGGGEVDET------------DKEEEPNAETTVVPS 681

ThHAK5 EPDEFERQFVHYLKEFIHHEYFISGGGGDVEETT-----------DKEEEPNIETTLVPM 687

OsHAK5 DPAEFESLVIENLKQFIHEESLYSQSSHSLEGE---------------SIKEIGGVTDPT 674

OsHAK1 EPREFAAFLVDGLKMFIQEESAFAPHQEMID-AAADDD--------DEAAARPRRSTSSA 695

HvHAK1 EPKEFAAFLVDRLKMFIQEESAFALVQDQEESGGAGDV--------SDALARPRRST--- 673

:. :* .: *: **:.* .

CcHAK1 VDSRISTSTRSI**H**SVHTPTAQSNRSSSRTQMVPPNASGQEEMQFVEKAKEQGVFYLLAEA 754

CaHAK1 VDSRISTSTRSI**Q**SVHTPTAQSNRSSSRTQMVPPNASGQEEMQFVEKAKEQGVFYLLAEA 754

LeHAK5 QQQQQVDST---TSPSTRSIQSNRSSSRIQVLHPNASGQEETQFIEKAKDQGVFYLLAEA 736

AtHAK5 --SNYVPSSGRIGSAHSSSSDKIRSGRVVQVQS----VEDQTELVEKAREKGMVYLMGET 735

ThHAK5 --SNSVASSGRVGSTHSSS-NKIRSGRVVQVQY----VEDHKDLVEKAREKGMVYLMGET 740

OsHAK5 SEVQDAMSSRNNSDQHTTEPRNG--------------CMDEIQSIHKEMGNGVVHLLGET 720

OsHAK1 VHSEEAIQAASSGRTTASSVQLQAGGE----PPAAMDVEEEKRLIDREVGRGVVYLMGEA 751

HvHAK1 VHSEEAVQGQARVSSHSASGRMSFH--------TSQAVEEEKQLIDREVERGMVYLMGEA 725

. . : :. :.: .*:.:*:.*:

CcHAK1 EVVAKKDSSFVKKAFVNYGYNFLRKNFRQGEKVMAIPQTRLLRVGMTYEV 804

CaHAK1 EVVAKKDSSFVKKAFVNYGYNFLRKNFRQGEKVMAIPQTRLLRVGMTYEV 804

LeHAK5 EVIAKQDSSFVKKGIINYGYSFLRKNFRQGEKVMAIPQTRLLRVGMTYEL 786

AtHAK5 EITAEKESSLFKKFIVNHAYNFLKKNCREGDKALAIPRSKLLKVGMTYEL 785

ThHAK5 EITAEKDSSLFKRFIVNHAYNFLKKNCREGDKALAIPRSKLLKVGMTYEL 790

OsHAK5 NVVAEPNADFLKKIIVDYVYNFIRKNFRQPEKITCVPHNRLLRVGMTYEI 770

OsHAK1 NVSAGPNSSILKRIAVNYIYTFLRKNLTEGHRALAIPNDQLLKVGITYEI 801

HvHAK1 NVTAEAKSSILKKIVVNHVYTFLRKNLTEGHKVLAIPKDQLLKVGITYEI 775

:: * .:.:.*: ::: *.*::** : .: .:*. :**:**:***:

**Fig. S2** Analysis of the sequence of the CcHAK1 transporter. Alignment of the amino acid sequence predicted for CcHAK1 with the amino acid sequences of the KUP/HAK/KT transporters of group I reported to date (CLUSTAL 2.1). Amino acid residues that are conserved among the HAK-type transporters of plants are shaded in gray; amino acid residues that differ in the CcHAK1 and CaHAK1 transporters are shown in red squares. The amino acid residue in the 356 position for CcHAK1 is shaded in black. The conserved sequence and the conserved amino acids in all HAK-type transporters in the first transmembrane segment is outlined and are shown in bold, respectively.

**Fig. S3** Tissue-specific expression of the *CcHAK1* gene in leaves and roots of habanero pepper. Pepper seedlings of 45 days old were transferred for ten days to Hoagland solution [1/5] containing 1.4 mM or 0.05 mM K^+^. The transcript levels were evaluated by semi-quantitative RT-PCR using the tubulin gene as a loading control.
